# PKM2, the “K^+^ sink” in the tumor interstitial fluid

**DOI:** 10.1093/procel/pwae036

**Published:** 2024-06-24

**Authors:** Wenjing Na, Wenfeng Zeng, Kai Song, Youwang Wang, Luoyang Wang, Ziran Zhao, Lingtao Jin, Ping Zhu, Wei Liang

**Affiliations:** Key Laboratory of Biomacromolecules (CAS), Institute of Biophysics, Chinese Academy of Sciences, Beijing 100101, China; College of Life Sciences, University of Chinese Academy of Sciences, Beijing 100864, China; Key Laboratory of Biomacromolecules (CAS), Institute of Biophysics, Chinese Academy of Sciences, Beijing 100101, China; College of Life Sciences, University of Chinese Academy of Sciences, Beijing 100864, China; College of Life Sciences, University of Chinese Academy of Sciences, Beijing 100864, China; Key Laboratory of Epigenetic Regulation and Intervention, Chinese Academy of Sciences, Beijing 100101, China; College of Life Sciences, University of Chinese Academy of Sciences, Beijing 100864, China; Key Laboratory of Epigenetic Regulation and Intervention, Chinese Academy of Sciences, Beijing 100101, China; School of Basic Medicine, Qingdao University, Qingdao 266071, China; Thoracic Surgery Department, National Cancer Center/National Clinical Research Center for Cancer/Cancer Hospital, Chinese Academy of Medical Sciences and Peking Union Medical College, Beijing 100021, China; Department of Molecular Medicine, University of Texas Health Science Center at San Antonio, TX 78229, USA; College of Life Sciences, University of Chinese Academy of Sciences, Beijing 100864, China; Key Laboratory of Epigenetic Regulation and Intervention, Chinese Academy of Sciences, Beijing 100101, China; Key Laboratory of Biomacromolecules (CAS), Institute of Biophysics, Chinese Academy of Sciences, Beijing 100101, China; College of Life Sciences, University of Chinese Academy of Sciences, Beijing 100864, China

## Abstract

**Graphical Abstract:**
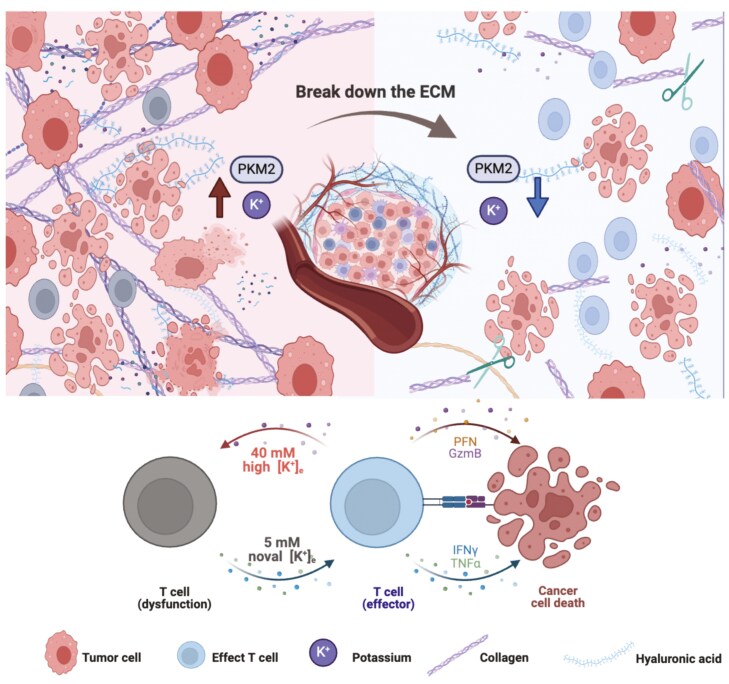

**Dear Editor,**

The microenvironment within a solid tumor is a complex entity comprising cellular components, encompassing tumor cells, fibroblasts, adipocytes, endothelial cells and immune cells, alongside noncellular components such as extracellular matrix (ECM) and tumor interstitial fluid (TIF) (Cox, 2021; Di Martino et al., 2021; Giussani et al., 2019; Poltavets et al., 2018). The genesis and progression of tumors hinge not solely on cancer cell-autonomous processes but intricately involve both cellular and noncellular constituents of the tumor microenvironment (TME) (Fukumura and Jain, 2007; Hanahan and Coussens, 2012; Joyce and Fearon, 2015). While earlier investigations into the tumor microenvironment primarily concentrated on the stromal and cellular elements of tumors (Henke et al., 2019), recent attention has been directed toward unraveling the complexities of TIF. TIF, a highly dynamic and versatile milieu, undergoes constant changes in its composition due to factors such as cell necrosis, tumor growth, and abnormal fluid outflow (Butler et al., 1975; Freitas et al., 1997; Wiig et al., 2010). Notably, TIF, in turn, exerts a profound influence on the activity and function of tumor-associated cells (Wagner and Wiig, 2015; Wiig et al., 2010). Despite the growing interest in TIF, its study remains in its infancy, with much left to be explored and understood.

Recent studies have brought to light a crucial aspect of TME—environmental factors, specifically potassium ions, have been identified as inhibitors of CD8^+^ T cell function, thereby tilting the immune landscape toward a suppressive state (Eil et al., 2016; Vodnala et al., 2019). This newly recognized tumor ion imbalance has emerged as a novel immunosuppressive factor, impeding the functionality of effector CD8^+^ T cells, fostering their exhaustion, and prompting the differentiation of tumor-associated macrophages (TAMs) into the M2 phenotype (Chen et al., 2022). The heightened accumulation of potassium ions at the tumor site is attributed to the release of necrotic tumor cells caused by nutrient imbalances. Yet, the mechanism underlying how potassium ions are retained in TIF without diffusing along the concentration gradient still remains elusive (Lunt et al., 2008). Unraveling the fundamental mechanisms governing potassium ion retention in TIF holds promise for providing fresh insights into the formation of immune-suppressive TME and may pave the way for innovative strategies to mitigate immune suppression, enhancing the efficacy of immunotherapeutic approaches in tumor treatments.

To substantiate the observed phenomenon of heightened potassium ions in TIF, we conducted a comprehensive analysis using tissue samples collected from 11 non-small cell lung cancer (NSCLC) patients, comprising both tumor and adjacent para-cancerous tissues. The results unequivocally demonstrated that the concentration of potassium ions in TIF ([K^+^]_e_) was markedly and exclusively elevated compared to the interstitial fluid of adjacent para-cancerous tissues (PIF) (Fig. 1A). To ascertain the universality of this finding, we established various tumor models, including B16F10, 4T1, MC38, TC-1, and A549 models. Across all tested tumor models, a consistent and significant increase in [K^+^]_e_ concentration in TIF was observed, while concentrations of sodium (Na^+^), calcium (Ca^2+^) or chloride (Cl^s^) ions remained unchanged (Fig. S1A). Notably, the heightened [K^+^]_e_ concentration was found to be independent of tumor sizes (Fig. S1B and S1C). This observation firmly establishes the existence of a high [K^+^]_e_ state within the TIF of solid tumors. Furthermore, we observed that high [K^+^]_e_ significantly inhibited the function and proliferation of CD8^+^ T cells (Fig. S2A–C), and suppressed the potential of naïve T cells to differentiate into T helper cells (including Th1, Th2, and Th17), while the differentiation into Treg cells appeared to be less sensitive to the high [K^+^]_e_ status as in TME (Fig. S2D and S2E). Moreover, under the high [K^+^]_e_ condition, tumor-associated macrophages (TAMs) tended to differentiate into protumor M2-type macrophages (Fig. S2F). The high-potassium environment constructs an immunosuppressive microenvironment. It is worth noting that restoration of K^+^ levels to the physiological level (5 mmol/L) partially revived the proliferative potential and tumor-killing ability of CD8^+^ T cells, indicating the reversible nature of the inhibitory impact imposed by high [K^+^]_e_ (Fig. S2A–C). The illustration of how potassium ions are retained in TIF will provide new insights into the formation of immunosuppressive TME.

**Figure 1.**
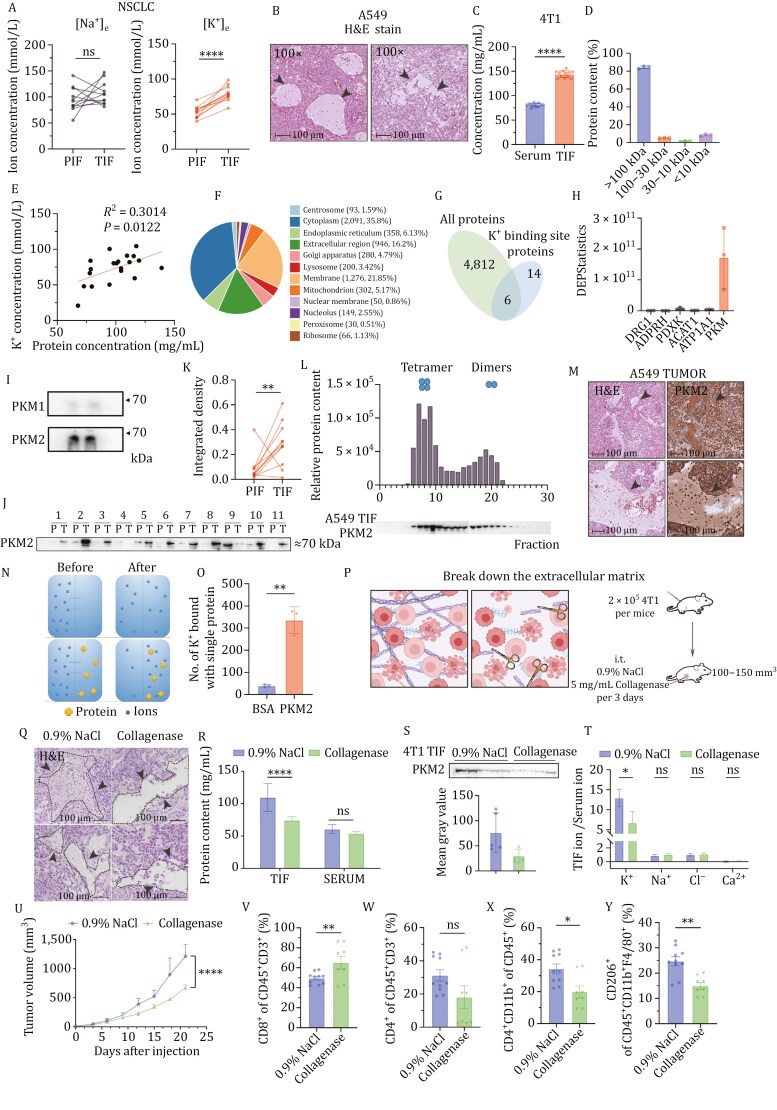
The PKM2 can specifically bind potassium ions in tumor interstitial fluid. (A) Sodium and Potassium ion concentrations in TIF and PIF of NSCLC patient samples, *n* = 11. (B) Representative H&E staining images of A549 tumor tissues (*n* = 7) (×100), scale bar = 100 μm. (C) Protein concentrations in TIF and serum of 4T1 bearing mice (*n* = 12). (D) Percentage of proteins with different molecular weight ranges in TIF of 4T1 tumors. (E) Correlation analysis between protein concentration and potassium ion concentrations in TIF. (F) The pie chart depicting protein localization in TIF form NSCLC patient samples by DIA proteomics, *n* = 3. (G and H) Number and relative content of proteins with potassium binding site in TIF form NSCLC patient samples, *n* = 3. (I) PKM2 as the main form present in TIF, *n* = 2. (J and K) PKM2 levels in TIF and PIF of NSCLC patient samples, *n* = 11. (L) The protein fractions with different molecular weights in A549 TIF were separated by molecular sieve. (M) Representative immunohistochemistry images of PKM2 location in A549 tumor tissues, *n* = 8, scale bar = 100 μm. (N and O) Schematic diagram of equilibrium dialysis and the quantification of the number of K^+^ bound with single protein *in vitro*, pH = 6.5, spheres representing potassium ions, crosses representing proteins. (P) Schematic diagram of tumor extracellular matrix and the enzymatic digestion therapy in 4T1 model. (Q) Representative H&E staining images tumor tissues with or without collagenase treatment (*n* = 4). (R) The protein concentration in TIF or serum (*n* = 5). (S) the western blot and quantification of PKM2 in TIF from the 4T1 model with or without collagenase treatment. (T) The ratio of ion concentrations in TIF over serum from the 4T1 model with or without collagenase treatment. (U) Tumor growth curves. (V–Y) The frequency of tumor infiltrating CD8^+^ T cells, CD4^+^ T cells, MDSC and TAM2in 4T1 model with or without collagenase treatment. Data in (A), (C), (K), (O), (R), (T), (U–Y) are represented as the mean ± SEM. Statistical analysis was performed using a two-tailed unpaired Student’s *t*-test or one-way ANOVA followed by *post hoc t*-tests. *P < 0.05; **P < 0.01; ****P < 0.001.

The heightened [K^+^]_e_ state in TIF primarily stems from extensive cellular necrosis, as illustrated in Fig. 1B where necrotic areas are indicated by arrows. This necrosis is a consequence of the paradoxical interplay between rapid cell proliferation and nutrient deprivation. The underlying mechanism of this elevation of atomic-sized [K^+^]_e_ in this context, rather than diffusing along concentration gradients outside the tissues, remains elusive. Numerous studies have supported the notion that diverse proteins exhibit unique ion-binding capabilities through specific metal-binding sites or distinctive higher-order structures, such as double-ion layer structures formed by proteins. In our investigations across various murine tumor models and tumor specimens from lung cancer patients, we observed a significant increase in protein content within the TIF compared to serum or PIF (Figs. 1C and S1D). Intriguingly, proteins with a molecular weight larger than 100 kDa were notably abundant in TIF, constituting ~80% of the total protein contents (Fig. 1D). Unlike normal tissues, the tumor stroma is densely populated with noncellular constituents like hyaluronic acids and collagens, establishing a matrix system akin to a “molecular barrier”. Consequently, macromolecules are sequestered within the solid tumor, leading to protein retention and an increase in pressure. Of particular note, we observed a pronounced positive correlation between protein concentration and [K^+^]_e_, while no significant correlation with other ions was discerned (Figs. 1E and S1E). As a result, we postulate that a specific protein within the TME may be responsible for the retention of potassium ions in TIF.

In our pursuit of identifying the key protein(s) responsible for potassium ion binding, we conducted Data-Independent Acquisition (DIA) proteomic profiling on TIF/PIF derived from NSCLC patient samples. Although the proteins and pathways involved in TIF and PIF were similar in nature, their content differed significantly (Fig. S3A–D). The proteomic analysis unveiled that a majority of TIF proteins originated from the cytoplasm (Fig. 1F), supporting the hypothesis that these proteins in TIF were released due to extensive cell necrosis. Through cross-comparison of the proteins with known potassium ion binding sites, six proteins were pinpointed in TIF: DRG1, ADPRH, PDXK, ACAT1, ATP1A1, and PKM (Fig. 1G and 1H). Among these proteins, PKM exhibited the highest abundance in TIF. Western blotting confirmed the presence of both PKM isoforms in TIF, with PKM2 being the predominant isoform in tumors (Fig. 1I) (Christofk et al., 2008). Analyzing TIF and PIF derived from NSCLC patients, we observed that PKM2 was more abundant in TIF than PIF in 9 out of 11 tested samples (Fig. 1J and 1K). Given that PKM2 predominately exists in the tetrameric form in cancerous cells but switches between dimmer and tetramer in healthy cells (Gavriilidou et al., 2018; He et al., 2022; Wang et al., 2015), we investigated whether the form of PKM2 in TIF influences potassium ion binging. Molecular sieve chromatography analysis determined that the majority of PKM2 in TIF existed in the tetrameric form, with an approximate molecular weight of 240 kDa in both 4T1 and A549 tumor TIF samples (Figs. 1L and S3E), supported by the native-page results (Fig. S3F). Additionally, immunohistochemistry (IHC) analysis further substantiated the co-localization of PKM2 with the TIF area (Fig. 1M). To quantify the amount of PKM2 in tumor stroma, a semi-quantitative western blotting-based method was employed, using a serially diluted purified PKM2 protein as the standard. and the concentration of PKM2 in TIF was determined to be in the range of 2–3 mg/mL (Fig. S3G).

To further validate the ion-binding capacity of PKM2, we established a prokaryotic expression system for PKM2 and purified the protein (Fig. S4A and S4B). The aggregation form and molecular weight of the purified PKM2 were characterized using native-page and static light scattering, confirming the presence of tetrameric PKM2 consistent with the PKM2 form found in TIF (Fig. S4C). Subsequently, an *in vitro* equilibrium dialysis system was constructed, as illustrated in Fig. 1N. In this system, the protein pool and ion pool were separated by a semi-permeable membrane with pores preventing substances with molecular weights larger than 10 kDa from diffusing across. After a period of equilibration, during which ions in the ion pool could freely diffuse due to the concentration gradient, the ion concentrations in both pools reached equilibrium. In the absence of protein–ion binding, the ion concentrations would become equally the same across the membrane. However, in the presence of specific protein–ion binding, the ion concentration in the protein pool would be higher than that in the ion pool. By comparing the rate of ion changes before and after equilibrium, the Number of Ion Bound with Single Protein could be calculated. Initially, we used albumin as a model protein, considering its high abundance in TIF (Table S1). The system reached complete equilibrium within 6–12 h (Fig. S4D and S4E), and therefore, 8 h were chosen as the detection time point to examine the binding of PKM2 or BSA to potassium ions at the same concentration (2 mg/mL). In comparison to BSA, PKM2 exhibited a stronger binding affinity to potassium ions (Fig. 1O), and the binding of chloride ions also increased correspondingly. We hypothesized that the protein might generate electrostatic interactions such as double-ion layers, leading to the attraction of ions with negative charges. Furthermore, we employed a Cas-9 strategy to knock down PKM2 in the 4T1 cell line. In the 4T1^PKM2-KD^ model, both PKM2 levels and [K^+^]_e_ in TIF were partially reduced (Fig. S5A–C), providing additional confirmation of the binding of PKM2 to potassium ions *in vivo*.

The tumor stroma, primarily composed of hyaluronic acids and collagens, prompted us to employ enzymatic strategies using collagenase and hyaluronidase to disrupt the stromal network (Fig. 1P and 1Q). Notably, collagenase significantly reduced protein concentrations in TIF (Fig. 1R), while hyaluronidase did not yield a similar reduction (Fig. S5D). This distinction may be attributed to their differential localization, with hyaluronic acids mainly present at the outer edges of the solid tumor, while collagens are distributed extensively within the internal regions, particularly in the interstitium. Consistent with the overall reduction in protein concentration, western blot analysis revealed a decrease in PKM2 (Fig. 1S). Furthermore, ion concentration measurements demonstrated that only [K^+^]_e_ was significantly reduced, aligning with our speculation (Fig. 1T). *In situ*, collagenase digestion significantly impeded tumor growth in immune-competent mice without the addition of other antitumor agents or treatments (Fig. 1U). Notably, the impact of collagenase digestion on tumor growth was not observed in nude mice (Fig. S5G). Immunotyping of the tumor revealed a remarkable enhancement in the immune profile of the collagenase-treated group, characterized by improved antitumor immunity (Fig. 1V–Y), a significant increase in CD8^+^ T cell proportion, a decrease in Treg proportion, and a marked reduction of MDSC levels. Conversely, treatment with hyaluronidase did not reduce [K^+^]_e_ or improve the immune status in TME (Fig. S5E and S5F).

Through equilibrium dialysis, we discovered that PKM2 can bind a substantial number of potassium ions, with each protein capable of binding 300–400 potassium ions. However, the number of binding ions in previous literature for this stable potassium ion binding site in PKM2 was considered much less (Schormann et al., 2019). This unexpected result lets us suspect a potential correlation between the capacity of ion binding and the overall structural change of the protein. To delve deeper into the binding mechanism of PKM2 to potassium ions (K^+^), we selected other members of group 1 elements, namely Na^+^ and Rb^+^, as control ions to conduct the comparative study, since they possess similar charge densities with each other but different hydrated radius (Fig. 2A). Initially, under pH 6.5 or 8.0, we aimed to identify the overall structural changes in PKM2 at different concentrations and ion states. Fluorescence emission spectroscopy results revealed that PKM2 exhibited higher selectivity to K^+^ at pH 6.5, undergoing more significant structural changes compared to Na^+^ or Rb^+^, while PKM2 maintained structural stability at pH 8.0 (Fig. 2B). And PKM2 underwent the maximum structural changes at 50 mmol/L [K^+^]_e_ under pH 6.5 (Fig. 2B), under which condition PKM2 was found to bind more potassium ions compared to that under pH 8.0 (Fig. 2C). This finding drove us to hypothesize that both pH and the ion concentration could result in structural changes of PKM2 which in turn affect the surface charge of the protein (namely to be more negatively charged) to attract more positive ions, like K^+^. To this end, we measured the zeta potential at 50 mmol/L ions under pH 6.5, the condition at which the maximum structural changes occurred. It was observed that more potassium ions entered the slipping layer, indicating that K^+^ had a comparatively greater potential to neutralize the negative charges of the PKM2 stern layer than Na^+^ and Rb^+^. In conclusion, compared to the other two monovalent cations, K^+^ penetrated the stable double-ion layer of PKM2 to a greater extent (Fig. 2D).

**Figure 2.**
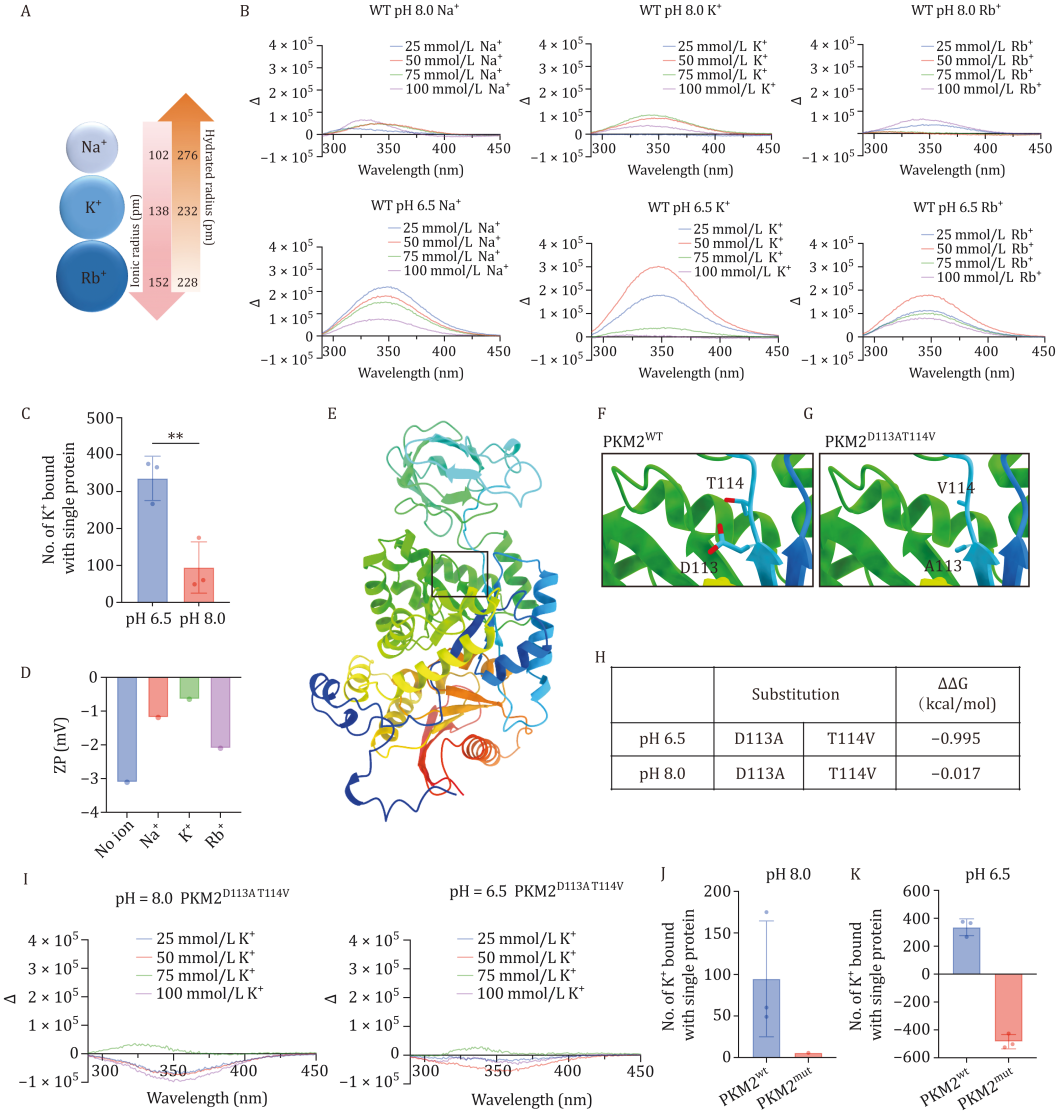
PKM2 selectively binds K^+^ through conformational changes and electrostatic interaction. (A) Ion radius and hydrated radius of Na^+^, K^+^, Rb^+^. (B) Fluorescence emission spectrum changes (Δ) of PKM2^WT.^ (C) The number of K^+^ bound with a single protein in different pH conditions at room temperature. (D) Zeta potential with different ions, pH = 6.5. (E–G) PKM2^D113AT114V^ structure by Alphafold2 and the mutant substitution. (H) The estimated relative binding free-energy difference calculations for residue mutation. (I) Fluorescence emission spectrum changes (Δ) of PKM2^D113AT114V^. (J) K^+^ bound with single protein of PKM2^WT^ and PKM2^D113AT114V^, pH = 8.0, time = 8 h, RT. (K) K^+^ bound with single protein of PKM2^WT^ and PKM2^D113AT114V^, pH = 6.5, time = 8 h, RT. Data in (C) are represented as the mean ± SEM. Statistical analysis was performed using a two-tailed unpaired Student’s *t*-test. **P < 0.01.

To further examine the relationship between structural changes and ion binding capacity, we mutated the known K^+^ binding sites in wild-type PKM2 to generate and purify the PKM2^D113AT114V^ mutant protein (Figs. 2E, 2F and S6A) (Schormann et al., 2019). *In silico* binding free-energy calculations further suggested that PKM2^D113AT114V^ mutant protein was more stable than PKM2^wild-type^ (WT) (Fig. 2G and 2H). Subsequently, employing fluorescence emission spectroscopy and circular dichroism spectroscopy, we found that, upon treatment with various concentrations of K^+^, the structural change in PKM2^D113AT114V^ was less sensitive than in PKM2^WT^ (Fig. S6B–G). Importantly, the binding ability of PKM2^D113AT114V^ to K^+^ was significantly reduced (Fig. 2I and 2J). These findings confirm that structural changes of PKM2 in the slightly acidic tumor microenvironment (pH 6.5) allow potassium ions to bind with PKM2 through electrostatic force and, consequently, be retained in the tumor stroma. Moreover, the binding of K^+^ induces significant structural changes, leading to more K^+^ binding—forming positive feedback at the protein structural level.

In the present study, we unveiled a novel mechanism contributing to the retention of potassium ions in the tumor interstitial fluid (TIF), primarily orchestrated by a key protein released from necrotic tumor cells—PKM2. The extensive necrosis of tumor cells at the tumor site leads to the release of PKM2 into the TIF. Intriguingly, extracellular PKM2 exhibits a unique ability to bind potassium ions (K^+^) independent of its enzymatic function. PKM2 selectively binds to K^+^ through electrostatic interactions, inducing significant structural changes in the protein and attracting more K^+^ to bind with PKM2. We term this phenomenon the “K^+^ sink”, which may explain the universality of acquired immunosuppression within TME since any antitumor treatment would lead to massive tumor cell necrosis and K^+^ sink would inevitably occur. In summary, we propose “K^+^ sink” in TIF may be one of the main causes of the overall immunosuppression within the tumor, and targeting ionic dynamics, such as *in situ* collagenase digestion method in the present study, could be a valuable approach to potentially augment the antitumor efficacy of current tumor immunotherapies. Further exploratory research is warranted to deepen our understanding of the underlying mechanisms.

## Supplementary Material

pwae036_suppl_Supplementary_Material

## Footnotes

We thank Dr. Junfeng Hao from the core facility-Center of Animal Research and Platform for Pathology Research at the Institute of Biophysics, Chinese Academy of Sciences for technical support in our immunohistochemistry study.

All the authors declare no competing interests.

This work was supported by the National Natural Science Foundation of China (81702823 to W.Z.), the Priority Research Program from the Chinese Academy of Sciences (XDB37010100 to P.Z.), and the Basic Research Program Based on Major Scientific Infrastructures of Chinese Academy of Sciences (JZHKYPT-2021-05 to P.Z.). The experimental protocols of this study were approved by the Animal Care and Use Committee of the Institute of Biophysics, Chinese Academy of Science, China (SYXK2021128), The use of tissues from patients with NSCLC was approved by the ethics committees at the Chinese Academy of Medical Sciences and Peking Union Medical College, Beijing, China (NCC2022C-702, from 8 June 2022), and all experiments were performed in accordance with relevant guidelines and regulations.

W.N., W.Z., and W.L. conceived the project and designed the experiments; W.N. and W.Z. conducted mouse and cell-related experiments; W.N., W.Z. and L.W. performed analyzed DIA-proteomics data; Z.Z. provided clinical samples; W.N. and K.S. purified PKM2; Y.W. conducted the molecular simulation and calculation; K.S. and L.J provided constructive advice to improve the research and manuscript preparation; W.N. and W.Z. analyzed the data and wrote the manuscript; P.Z. and W.L. jointly supervised the project.

All data are available in the main text or the Supplementary materials.
