# Supplementary material for: PKM2, the “K+ sink” in the tumor interstitial fluid
Source: Protein Cell. 2024 Jun 24;16(4):300–6. doi: 10.1093/procel/pwae036 (PMC12053478; doi:10.1093/procel/pwae036)

Supplemental Figure Legends

Figure S1. High potassium in TIF hardly correlates with tumor sizes.

(A) The ratio of ion concentrations in TIF over serum of different tumor models, including B16F10, 4T1, MC38, TC1, ADR and A549 (n=6 of each model). **(B-C)** Ion concentrations in the tumor interstitial fluid with different sizes (n=5 of each model). **(D)** Protein concentration in TIF and PIF of NSCLC patient samples (n=11). **(E)** Correlation analysis between protein concentration and Na^+/Cl^- concentration in TIF. Data in (D) are represented as the mean ± SEM. Statistical analysis was performed using a two-tailed unpaired Student's *t*-test. *P < 0.05.

Figure S2. Immunosuppression due to high potassium is reversible.

(A) *In vitro* $CD8^+$ T cell proliferation can be recovered by re-incubating with physiological $[K^+]_e$ level. **(B-C)** Co-culture system for *in vitro* tumor cytotoxicity assay. **(D-F)** The effect of different $[K^+]_e$ on the differentiation potency of naive $CD4^+$ T cells, including T helper cells, Tregs, and tumor-associated macrophages (TAM). Data in (A), (C), (D), (E) and (F) are represented as the mean ± SEM. Statistical analysis was performed using a two-tailed unpaired Student's *t*-test. *P < 0.05; **P < 0.01; ***P < 0.005; ****P < 0.001.

Figure S3. PKM2 quantification in tumor interstitial fluid（TIF）.

(A-B) The enrich analysis of GO and KEGG from TIF by DIA proteomics. **(C)** The pie chart showing the localization of proteins in NSCLC patient TIF samples by DIA proteomics. **(D)** The volcano chart showing differential expression of proteins between PIF and TIF. **(E)** Molecular sieve and the protein expression of different fractions in 4T1 TIF. **(F)** The Native PAGE gel showing the presence form of PKM2 in TIF of 4T1 model. **(G)** *In vitro* establishment of standard curve using a serious of concentrations of purified PKM2 for further quantification use.

Figure S4. The expression and purification of PKM2 and the establishment of equilibrium dialysis system.

(A) The design and construction of PKM2 expression plasmid by SnapGene. **(B-C)** SDS-PAGE gel and native gel results indicate purified PKM2. **(D)** The static light scattering analysis on purified PKM2 at different pH conditions. **(E)** *In vitro* equilibrium dialysis system shows the ions reach equilibrium without protein. **(F)** *In vitro* equilibrium dialysis system shows the ions reach equilibrium with 2 mg/mL BSA.

Figure S5. Lowering $[K^+]_e$ facilitates the restoration of anti-tumor immunity.

(A-C) The expression level of PKM2 and ion (Na^+, K^+) concentrations in TIF were detected in 4T1 model with or without PKM2 knockout (n=5). **(D)** The protein concentration in TIF or serum of 4T1 model with or without hyaluronidase (i.t.) treatment. **(E)** The ratio of ion concentrations in TIF over serum of 4T1 model with or without hyaluronidase treatment. **(F)** Tumor immunological profiling including $CD8^+$ T cells, $CD4^+$ T cells, MDSC and TAM2 (n=5). **(G)** Enzymatic treatment of 4T1 model on nude mice (n=3). **(H)** Representative H&E staining images tumor tissues with or without collagenase or hyaluronidase treatment, scale bar=100 μm (n=4).

Figure S6. Purification and structural changes of $PKM2^{D113AT114V}$ and $PKM2^{WT}$.

(A) The SDS-PAGE gel showing purified mutant $PKM2^{D113AT114V}$ protein. **(B-G)** Circular dichroism analysis on $PKM2^{D113AT114V}$ and $PKM2^{WT}$ with different ions.

Supplemental Tables

Table S1. Differentiation conditions for each CD4$^+$/CD8$^+$ T cell subset

	**Naïve CD4$^+$ T cell**					**CD8$^+$ T cell**
	**Th0**	**Th1**	**Th2**	**Treg**	**Th17**	
**Cytokines (ng/mL)**						
**rmIL-2 (U/mL)**<br>(PEPROTECH, #96-212-12)	30	30	30	30		
**rmIL-12**<br>(PEPROTECH, #96-210-12)		15				
**rmIL-4**<br>(PEPROTECH, #96-214-14)			10			
**rmIL-6**<br>(PEPROTECH, #96-216-16)					20	
**rmTGF-b**<br>(R&D, #7666-MB-005)				15	3	
**Antibodies (ug/mL)**						
**a-CD3 (2C11)**<br>(Invitrogen, #16-0031-86)	1	1	1	1	2	1
**a-CD28 (37.51)**<br>(Invitrogen, #16-0281-85)	1	1	1	1	1	0.5
**a-IL-4 (11B11)**<br>(Invitrogen, #16-7041-85)		5		5	5	
**a-IFNg (XMG1.2)**<br>(Invitrogen, #16-7311-85)			5	5	5	

SUPPLEMENTARY 1

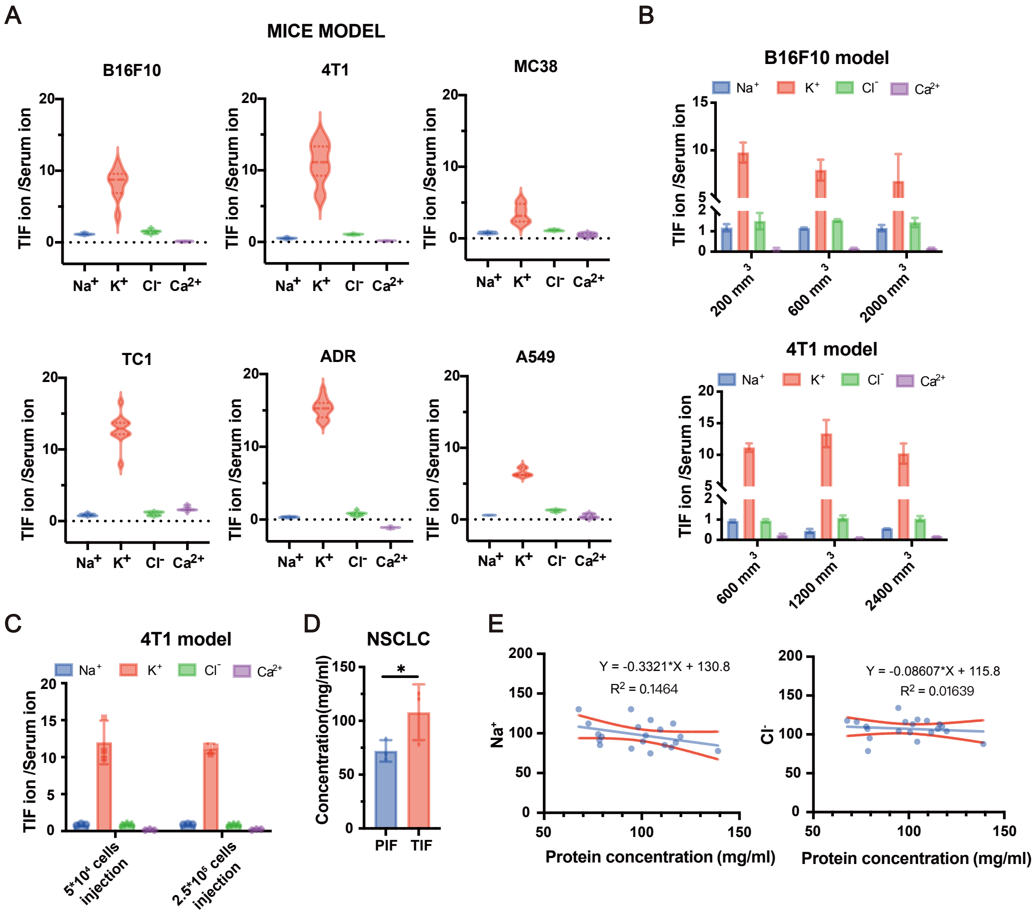

SUPPLEMENTARY 2

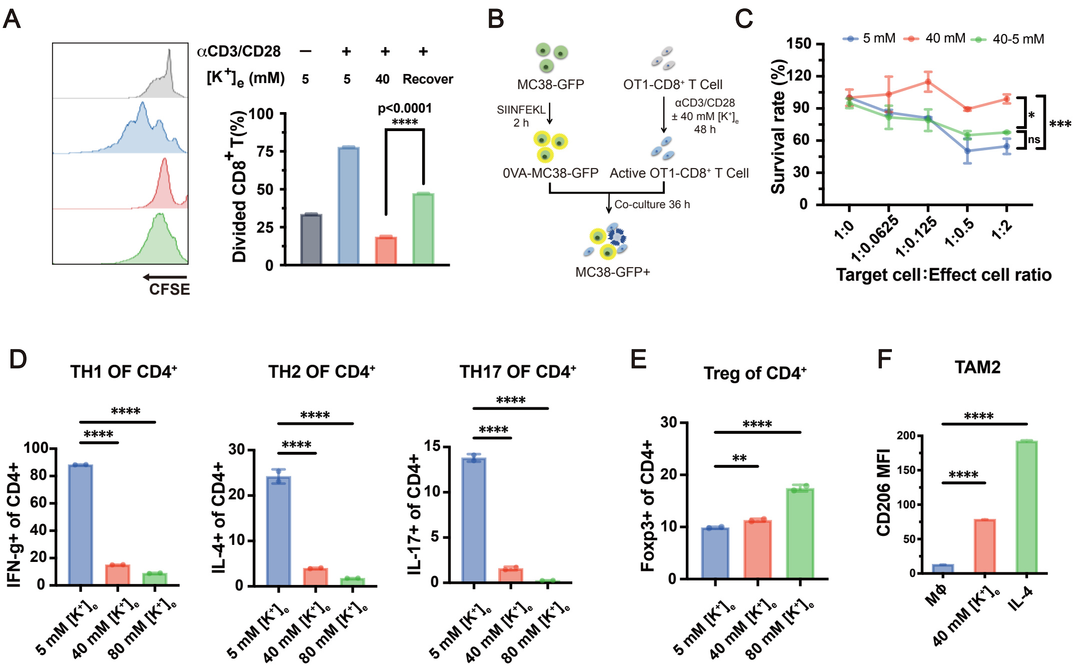

SUPPLEMENTARY 3

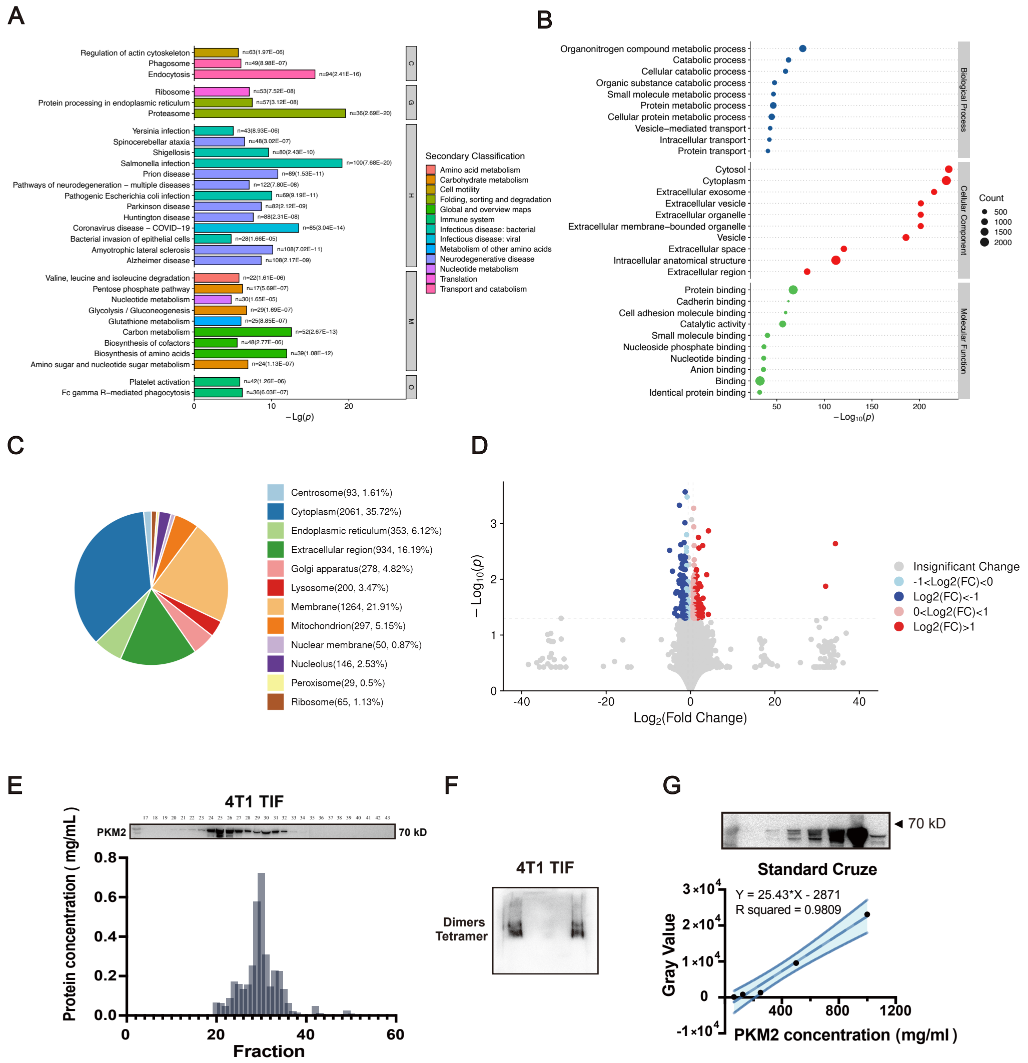

SUPPLEMENTARY 4

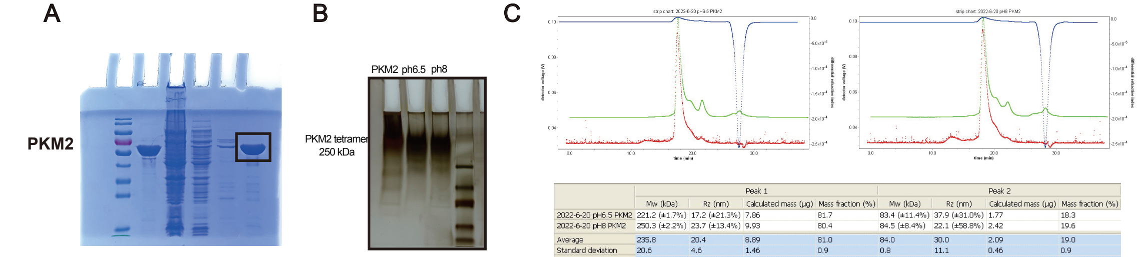

	Peak 1				Peak 2			
	Mw (kDa)	Rz (nm)	Calculated mass (μg)	Mass fraction (%)	Mw (kDa)	Rz (nm)	Calculated mass (μg)	Mass fraction (%)
2022-6-20 pH6.5 PKM2	221.2 (±1.7%)	17.2 (±21.3%)	7.86	81.7	83.4 (±11.4%)	37.9 (±31.0%)	1.77	18.3
2022-6-20 pH8 PKM2	250.3 (±2.2%)	23.7 (±13.4%)	9.93	80.4	84.5 (±8.4%)	22.1 (±58.8%)	2.42	19.6
Average	235.8	20.4	8.89	81.0	84.0	30.0	2.09	19.0
Standard deviation	20.6	4.6	1.46	0.9	0.8	11.1	0.46	0.9
% Standard deviation	8.7	22.4	16.44	1.1	0.9	37.1	22.11	4.7
Minimum	221.2	17.2	7.86	80.4	83.4	22.1	1.77	18.3
Maximum	250.3	23.7	9.93	81.7	84.5	37.9	2.42	19.6

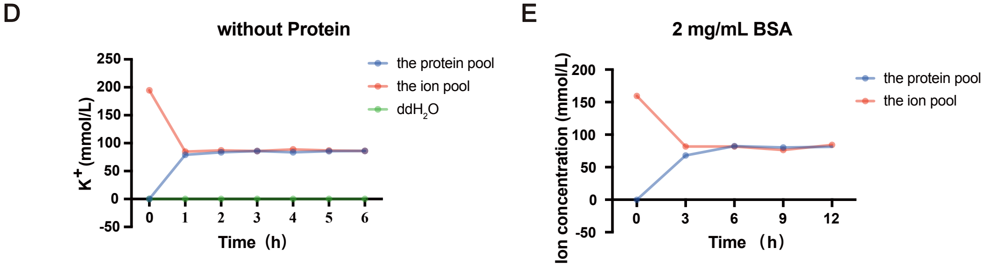

SUPPLEMENTARY 5

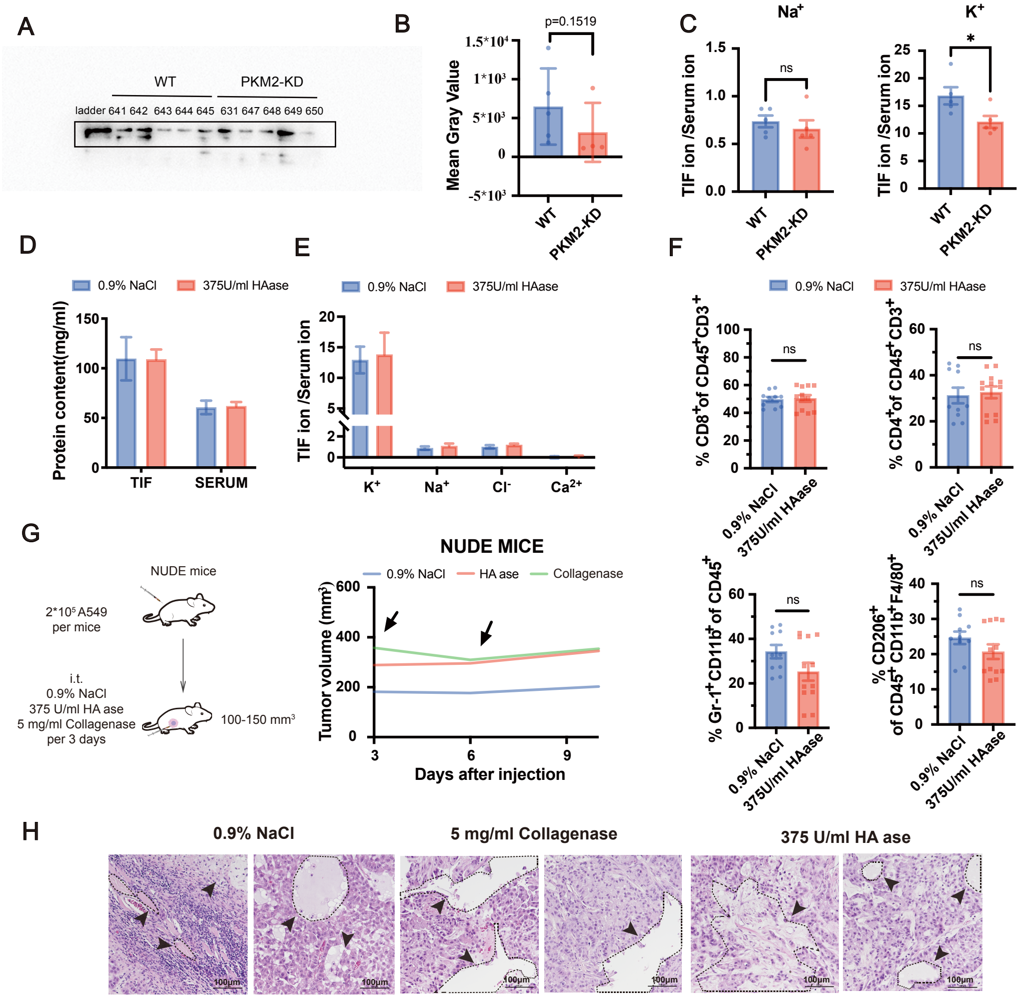

SUPPLEMENTARY 6

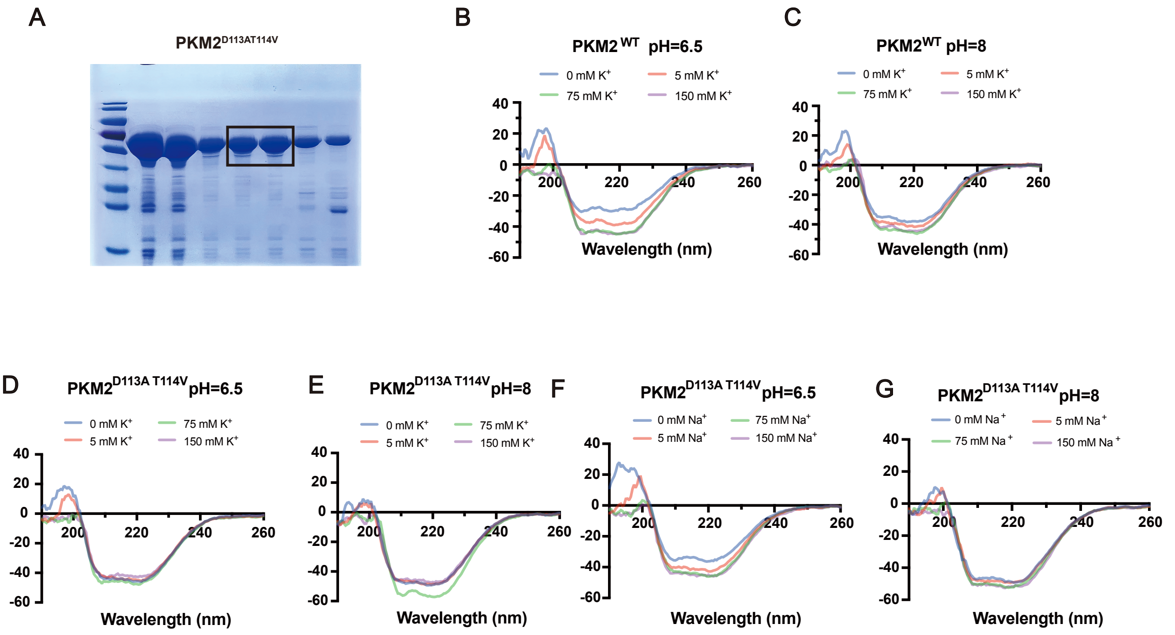

Supplement: pwae036_suppl_Supplementary_Material [file pwae036_suppl_supplementary_material.pdf]
